# Incidence and Factors Associated With Recurrent Pericarditis in Lupus

**DOI:** 10.1001/jamanetworkopen.2024.61610

**Published:** 2025-02-25

**Authors:** Yoo Jin Kim, Jana Lovell, Alaa Diab, Laurence S. Magder, Daniel Goldman, Michelle Petri, Andrea Fava, Luigi Adamo

**Affiliations:** 1 Department of Medicine, School of Medicine, Johns Hopkins University, Baltimore, Maryland; 2 Center for Cardiac Immunology, Division of Cardiology, Department of Medicine, Johns Hopkins University, Baltimore, Maryland; 3 Department of Epidemiology and Public Health, University of Maryland School of Medicine, Baltimore; 4 Division of Rheumatology, Department of Medicine, Johns Hopkins University, Baltimore, Maryland

## Abstract

**Importance:**

Pericarditis is the most common cardiac manifestation of systemic lupus erythematosus (SLE) and is known to recur among patients. However, the prevalence of and risk factors associated with recurrent pericarditis in patients with SLE were unknown.

**Objective:**

To investigate the frequency of and risk factors associated with the recurrence of pericarditis in patients with SLE.

**Design, Setting, and Participants:**

This cohort study was a retrospective analysis of a well-characterized, single-center prospective cohort of a diverse group patients with SLE treated at a tertiary medical center and enrolled between 1988 and 2023. Patients diagnosed with pericarditis among those enrolled in the Hopkins Lupus Cohort were included. Data were analyzed from April 2023 and May 2024.

**Main Outcomes and Measures:**

Recurrence of pericarditis was assessed. The Safety of Estrogens in Systemic Lupus Erythematosus National Assessment revision of the SLE Disease Activity Index (SELENA-SLEDAI) was used to define pericarditis. Clinical information was examined for all follow-up encounters after the first episode of pericarditis. Episodes that occurred at least 6 weeks after the first recorded episode were defined as *recurrent*.

**Results:**

Of 2931 patients within the Hopkins Lupus Cohort, 590 patients had a history of pericarditis (257 patients aged <30 years at first episode [43.6%]; 535 women [90.5%]; 303 Black [51.4%] and 253 White [42.9%]). In 21 patients (3.6), the diagnosis of pericarditis was confirmed via electrocardiogram or dedicated imaging, with 100% concordance between clinical and database diagnoses. During a median (IQR) follow-up of 6.7 (2.5-13.6) years and a total of 5277 years of follow-up, 120 patients (20.3%) experienced recurrent pericarditis (recurrence rate = 0.053 recurrences; 95% CI, 0.047-0.059 recurrences per person-year of follow-up). Among patients with recurrence, most patients (61 individuals [50.8%]) experienced only 1 recurrence, whereas 59 patients (49.2%) had 2 or more recurrences. In multivariable analysis, factors associated with recurrence included younger age (≥60 vs <40 years: rate ratio [RR], 0.11; 95% CI, 0.04-0.32), treatment with prednisone (≥20 mg vs 0 mg: RR, 1.99; 95% CI, 1.17-3.40), active SLE disease (SLEDAI ≥3 vs 0: RR, 1.55; 95% CI, 1.21-2.00), and time since initial episode (3-10 years vs <1 year: RR, 0.32; 95% CI, 0.20-0.52).

**Conclusions and Relevance:**

In this study, recurrence was more likely within 1 year of the onset of pericarditis and among younger patients, those with uncontrolled disease, and those receiving oral prednisone therapy, with a dose-dependent association. These findings may set the basis for future studies to define optimal treatment for recurrent pericarditis in patients with SLE and suggest that oral corticosteroids should be avoided when treating pericarditis in patients with SLE.

## Key Points

**Question:**

Among patients with systemic lupus erythematosus (SLE), what are the frequency and associated risk factors for the recurrence of pericarditis, the most common cardiac manifestation of SLE?

**Findings:**

In this cohort study of 590 patients with a history of pericarditis, 20.3% of patients experienced recurrence, among whom most patients (50.8%) experienced only 1 recurrence. In multivariable analysis, factors associated with recurrence included younger age, treatment with oral prednisone, active SLE disease, and time since the initial episode.

**Meaning:**

This study found that recurrence of pericarditis was more likely to occur within the first year since the initial diagnosis of pericarditis, among younger patients, and among those with severe SLE; additionally, findings suggest that oral prednisone therapy should be avoided when treating pericarditis given its association with recurrence.

## Teaser

This cohort study investigates the incidence of recurrent pericarditis among patients with a history of systemic lupus erythematosus (SLE) and factors associated with recurrence.

## Introduction

Pericarditis, defined as inflammation of the serosal sac that surrounds the myocardium, is the most common cardiac manifestation of systemic lupus erythematosus (SLE), and approximately 20% of patients with SLE experience pericarditis.^1,2,3^ Patients can experience a spectrum of symptoms, ranging from mild chest pain exacerbated by lying flat and improved by leaning forward to debilitating symptoms of severe chest pain and dyspnea. Importantly, pericarditis is associated with complications that include recurrence, myocarditis, and pericardial effusion.

In the general population, pericarditis recurs in approximately 30% of cases.^4^ Despite the significant rate of recurrence, mechanisms predisposing a subset of individuals to recurrence remain unclear. Caforio et al^5^ demonstrated the presence of serum antiheart and anti–intercalated-disk autoantibodies in patients with recurrent pericarditis. Furthermore, multiple clinical trials have demonstrated the efficacy of immunosuppressive agents, such as corticosteroids, in treating recurrent pericarditis, suggesting an immune-mediated mechanism.^5,6,7,8^ Pivotal studies from 2005 to 2023^7,8,9,10,11,12,13,14^ further support this hypothesis, particularly implicating interleukin 1, a proinflammatory cytokine of the innate immune system, in the pathophysiology of recurrent pericarditis. Inhibitors targeting this pathway have proven effective in treating idiopathic recurrent pericarditis across multiple randomized clinical trials.

Given the broad immune dysregulation associated with SLE, patients may face increased risk for recurrence owing to possible overlapping immune-mediated mechanisms. However, to our knowledge, the rate of recurrence of pericarditis in patients with SLE and the association of risk factors and various treatments with its recurrence have not been characterized. To address this knowledge gap, we analyzed the Hopkins Lupus Cohort, a large and well-characterized longitudinal cohort of patients with SLE.

## Methods

### Patients

The Hopkins Lupus Cohort is a single-center, longitudinal, prospective cohort of 2931 patients whose diagnosis of SLE is confirmed before study enrollment.^15^ Patients were enrolled at a single tertiary medical center from 1988 to 2023. The Hopkins Lupus Cohort is approved yearly by the Institutional Review Board of the Johns Hopkins University School of Medicine. All patients provided consent to enter the cohort. Patient history, laboratory testing, and clinical information relevant to the classification of SLE and the Systemic Lupus International Collaborating Clinics/American College of Rheumatology Damage Index are recorded at the time of cohort entry.^16^ Race was self-reported. Patients were asked to choose from among African American, Asian, White, and other race; this information was collected as part of the demographic data collected in our registry. We combined Asian and other categories owing to low population size. Patients in the cohort are followed up quarterly at a minimum, and clinical information and laboratory testing are updated at subsequent clinical visits. We identified patients who had a history of pericarditis at the time of cohort entry or a diagnosis of pericarditis during cohort participation using the criteria described subsequently. This study is reported following the Strengthening the Reporting of Observational Studies in Epidemiology (STROBE) reporting guideline.

### Diagnostic Criteria for Pericarditis

Pericarditis was diagnosed using the Safety of Estrogens in Lupus Erythematosus National Assessment–SLE Disease Activity Index (SELENA-SLEDAI), a validated tool in the assessment of SLE clinical activity.^17,18,19^ For the diagnosis of pericarditis, 1 of the following criteria needed to be present: pericardial pain, auscultation of pericardial rub, presence of pericardial effusion on imaging, or electrocardiogram (ECG) confirmation.^17,20^ Clinical information was examined for all follow-up encounters after the first episode of pericarditis. *Recurrent* pericarditis was defined as pericarditis that occurred at least 6 weeks after the first recorded episode. Multiple recurrent episodes were identified if pericarditis occurred at least 6 weeks apart. Patients with SLE flares that included pericarditis were reevaluated more often than the minimum required quarterly evaluations (eg, within 6 weeks) to escalate baseline immunosuppressant therapy as needed and discuss symptomatic response to treatment of the SLE flare.

### Statistical Analysis

The analysis was based on cohort observations that occurred after the patient’s first episode of pericarditis. For patients with a history of pericarditis prior to cohort entry, the time to recurrence was calculated from the date of cohort entry to the first episode documented during the cohort study, and all cohort follow-up encounters were included. Cohort data were reformatted into a dataset with 1 record for each person-month of follow-up. Each person-month record contained the information on the patient’s current treatments and most recent clinical findings. This file was used to calculate the rate of recurrence per person-month given various patient characteristics or exposures. For ease of interpretation, rates per person-month were converted to rates per person-year of follow-up. Rate ratios (RRs), 95% CIs, and *P* values were calculated using pooled logistic regression (a form of discrete survival analysis allowing for time-varying factors) as implemented using generalized estimating equations to account for some participants experiencing more than 1 recurrence.^21^ Significance was defined as a 2-sided *P* value < .05. Analyses were performed using SAS statistical software version 9.4 (SAS Institute). Data were analyzed from April 2023 and May 2024.

## Results

### Patient Characteristics

Among 2931 patients in the Hopkins Lupus Cohort, 590 patients with a history of pericarditis were included in the analysis (257 patients aged <30 years at first episode [43.6%]; 535 women [90.5%]; 303 Black [51.4%] and 253 White [42.9%]) (Table 1). Of these patients, 451 patients (76.4%) experienced their first episode of pericarditis prior to cohort entry, and the remaining 139 patients (23.6%) experienced their first episode while in the cohort. The median (IQR) duration of cohort follow-up observed after the initial acute pericarditis episode was 6.7 (2.5-13.6) years.

**Table 1.** Patient Demographic and Clinical Characteristics

Characteristic	Patients, No. (%) (N = 590)
Age at first episode of pericarditis, y	
<30	257 (43.6)
30-44	208 (35.3)
45-59	102 (17.3)
≥60	23 (3.9)
Sex	
Female	535 (90.7)
Male	55 (9.3)
Race	
Black	303 (51.4)
White	253 (42.9)
Other^a^	34 (5.8)
Onset of pericarditis	
<1990	106 (18.0)
1990-1999	165 (28.0)
2000-2009	215 (36.4)
≥2010	104 (17.6)
Duration of follow-up after onset of pericarditis, person-years	
<5	260 (44.1)
5-10	115 (19.5)
10-15	91 (15.4)
15-20	55 (9.3)
≥20	69 (11.7)
Recurrent episodes of pericarditis	
1	61 (10.3)
2-4	46 (7.8)
5-12	13 (2.2)

^a^ Other included Asian and other race.

### Overall Rate of Recurrence

We observed a total of 5277 years of follow-up, during which there were 278 recurrences of pericarditis, for a rate of 0.053 recurrences (95% CI, 0.047-0.059 recurrences) per person-year of follow-up. These events occurred in 120 different patients (20.3%), among whom 59 patients (49.2%) experienced more than 1 episode of recurrence and 61 patients (50.8%) experienced 1 recurrence.

### Diagnostic Criteria for Pericarditis

Patients were diagnosed with pericarditis based on SELENA-SLEDAI criteria. Most patients were diagnosed based on symptomatic reports of pericardial chest pain (567 patients [96.1%]). ECG or imaging studies were performed on a small subset of patients: ECG was conducted in 1 patient (0.2%), while transthoracic echocardiogram or computed tomography imaging findings demonstrated a pericardial effusion in 19 patients (3.2%) and 1 patient (0.2%), respectively. When diagnostic data were collected in conjunction with symptomatic reports of chest pain, the clinical diagnosis of pericarditis was confirmed 100% of the time (in all 21 patients).

### Risk Factors Associated With Recurrent Pericarditis

Demographic, clinical, serologic, and treatment characteristics were studied in univariate analysis to determine their association with recurrence among patients with pericarditis (Table 2). Sex was not associated with a change in recurrence rates. Black race demonstrated an RR of 1.72 (95% CI, 0.99-2.97) for risk of recurrent pericarditis, and older age was associated with decreased risk of recurrent pericarditis (ages 40-49 years: RR, 0.58; 95% CI, 0.37-0.90 or ages 50-59 years: RR, 0.25; 95% CI, 0.12-0.53 vs ages 18-39 years). Recurrence was more likely to occur within the first year from onset, after which the likelihood of recurrence decreased (eg, 1-3 years vs <1 year: RR, 0.50; 95% CI, 0.32-0.79).

**Table 2.** Rates of Recurrent Pericarditis

Characteristic	Events, No.	Rate, per person-year	RR (95% CI)
Demographic characteristics			
Age group, y			
18-39	171	0.085	1 [Reference]
40-49	74	0.054	0.58 (0.37-0.90)
50-59	28	0.025	0.25 (0.12-0.53)
≥60	5	0.006	0.05 (0.01-0.18)
Sex			
Female	260	0.054	1 [Reference]
Male	18	0.038	0.43 (0.11-1.69)
Race			
Black	172	0.061	1.72 (0.99-2.97)
White	97	0.045	1 [Reference]
Other	9	0.032	0.90 (0.34-2.34)
Year of onset of pericarditis			
Before 2000	71	0.082	1 [Reference]
2000-2009	98	0.055	0.59 (0.39-0.89)
After 2010	109	0.042	0.36 (0.22-0.61)
Clinical characteristics			
Time from initial episode, y			
<1	48	0.212	1 [Reference]
1-3	59	0.105	0.50 (0.32-0.79)
3-10	91	0.048	0.21 (0.14-0.32)
≥10	80	0.031	0.12 (0.06-0.23)
Most recent past clinical findings			
Disease activity, SLEDAI score			
0	52	0.032	1 [Reference]
1-3	58	0.043	1.32 (0.92-1.89)
3-10	91	0.048	0.21 (0.14-0.32)
≥10	80	0.031	0.12 (0.06-0.23)
C3 level			
Low	187	0.051	0.76 (0.52-1.09)
Normal	79	0.075	1 [Reference]
C4 level			
Low	190	0.048	0.54 (0.37-0.78)
Normal	75	0.098	1 [Reference]
Anti-dsDNA			
Seropositive	138	0.088	2.05 (1.40-2.99)
Seronegative	127	0.041	1 [Reference]
Various conditions			
Anti-SM			
Seropositive	198	0.053	1.06 (0.65-1.72)
Seronegative	75	0.049	1 [Reference]
Proteinuria			
History	123	0.041	0.58 (0.37-0.89)
No history	146	0.069	1 [Reference]
Nephrotic syndrome			
History	34	0.028	0.47 (0.27-0.82)
No history	242	0.06	1 [Reference]
Pulmonary fibrosis			
History	50	0.063	1.29 (0.55-3.02)
No history	227	0.051	1 [Reference]
Pulmonary hypertension			
History	26	0.028	0.42 (0.19-0.92)
No history	252	0.058	1 [Reference]
Hemolytic anemia			
History	33	0.038	0.76 (0.44-1.30)
No history	242	0.055	1 [Reference]
Lymphadenopathy			
History	107	0.047	0.70 (0.37-1.34)
No history	158	0.053	1 [Reference]
Raynaud syndrome			
Present	131	0.04	0.53 (0.27-1.07)
Not present	146	0.074	1 [Reference]
Current treatment characteristics			
Prednisone, mg/d			
0	98	0.045	1 [Reference]
1-9	79	0.052	1.54 (0.85-2.79)
10-19	45	0.076	2.23 (1.25-4.00)
≥20	216	0.063	3.92 (2.31-6.63)
Hydroxychloroquine use			
Yes	216	0.063	1.04 (0.70-1.53)
No	62	0.046	1 [Reference]
Immunosuppressant use			
Yes	112	0.058	0.92 (0.62-1.36)
No	166	0.059	1 [Reference]

Abbreviations: dsDNA, double-stranded DNA; RR, rate ratio; SLEDAI, Systemic Lupus Erythematosus Disease Activity Index; SM, smooth muscle.

We investigated the association of SLE disease activity with the risk of recurrence. Patients with active lupus (SLEDAI scale score ≥3) experienced significantly increased rates of recurrence (RR, 2.34; 95% CI, 1.65-3.30). Pericarditis is a component of the SLEDAI scale, and thus to further evaluate this observation, we repeated the analysis removing pericarditis from the SLEDAI score. This sensitivity analysis demonstrated a persistent positive association between a SLEDAI score of 3 or greater and recurrence of pericarditis (RR, 1.87; 95% CI, 1.33-2.64) (eTable 1 in Supplement 1), thus confirming that systemic SLE activity beyond the pericardium was associated with a risk of recurrence. Patients with kidney involvement, such as proteinuria or nephrotic syndrome, and those with pulmonary hypertension had decreased recurrence rates (Table 2). Hypocomplementemia was associated with increased rates of recurrence, with normal vs decreased levels of C4 associated with decreased risk (RR, 0.54; 95% CI, 0.37-0.78) while results were not significant for C3 levels (RR, 0.76; 95% CI, 0.52-1.09). Seropositivity for double-stranded DNA antibodies was associated with increased recurrence (RR, 2.05; 95% CI, 1.40-2.99).

Finally, therapies of patients with SLE who had recurrent pericarditis were assessed. Treatment with oral prednisone at any dose was associated with increased rates of recurrence. Furthermore, a positive, dose-dependent association with recurrence was observed (1-9 mg daily: RR, 1.54; 95% CI, 0.85-2.79; 10-19 mg daily: RR, 2.23; 95% CI, 1.25-4.00; ≥20 mg daily: RR, 3.92; 95% CI, 2.31-6.63). The use of hydroxychloroquine or other immunosuppressants was not associated with recurrence, and rates of pericarditis were lower after the year 2000 (Table 2).

To gain further insights into our findings, we performed a multivariable logistic regression model incorporating covariates identified as significant in univariate analyses (Table 2). The model identified younger age (eg, ≥60 vs <40 years: RR, 0.11; 95% CI, 0.04-0.32), time from first episode (eg, 3-10 years vs <1 year: RR, 0.32; 95% CI, 0.20-0.52), daily prednisone use (≥20 mg vs 0 mg: RR, 1.99; 95% CI, 1.17-3.40), and active disease (SLEDAI ≥3 vs 0: RR, 1.55; 95% CI, 1.21-2.00) as independent factors associated with recurrent pericarditis (Figure). Other variables found to be associated with recurrence in the univariate analyses (low C4 levels, anti–double-stranded DNA antibodies, proteinuria, and history of pulmonary hypertension) were not associated with recurrence after adjustment for other variables in the multivariable model (Figure). When the multivariable logistic regression model was repeated incorporating an SLEDAI score calculated without pericarditis, the odds ratio for high SLEDAI score and recurrent pericarditis did not reach our predefined level of significance (1.24; 95% CI, 0.96-1.61) (eTable 2 in Supplement 1).

**Figure.**
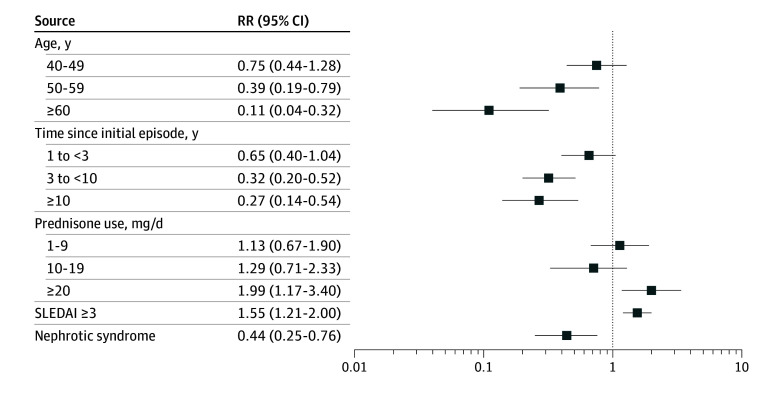
Multivariate Analyses of Factors Associated With Recurrent Pericarditis Reference groups were ages 18 to 39 years for age, less than 1 year for time since initial episode, and 0 mg/day for prednisone use. RR indicates rate ratio; SLEDAI, Systemic Lupus Erythematosus Disease Activity Index.

## Discussion

In this cohort study, we present a prospective analysis of recurrent pericarditis among patients with SLE. The rate of recurrent pericarditis among patients with SLE was 0.053 recurrences per year (120 of 590 patients [20.3%]). We found that whereas active SLE and younger age were factors associated with recurrence, systemic involvement beyond the pericardium, such as the renal system (eg, nephrotic syndrome or proteinuria) and pulmonary hypertension, were associated with decreased recurrence. Importantly, prednisone use was associated with an increased risk of disease recurrence. These findings expand our understanding of lupus pericarditis, underscore the need to reconsider using oral prednisone to treat SLE flares involving the pericardium, and identify the need for further investigation of treatments for lupus pericarditis.

SLE is a complex disease characterized by broad immune dysregulation that is considered the result of autoantibody production from irregularly activated and differentiated B lymphocytes and abnormal interferon activity.^22^ Similarly, the pathogenesis of recurrent pericarditis in the general population has been presumed to be immune mediated.^8^ Given the increased potential for autoimmunity among patients with SLE, we hypothesized that patients with SLE may experience an increased rate of recurrence after an acute episode of pericarditis. Surprisingly, we found that the rate of pericarditis recurrence among patients with SLE (20.3%) was lower than that reported in the general population (approximately 33%).^4,20^

Prior studies have examined risk factors associated with acute pericarditis among patients with SLE. Ryu et al^3^ reported factors that included African American race, anti–smooth muscle antibodies, anti–double-stranded DNA antibodies, hemolytic anemia, proteinuria, and Raynaud phenomenon. However, to the best of our knowledge, there have been no studies to date that have investigated risk factors associated with recurrence. We found that increased SLEDAI scores, younger age at disease onset, recurrence within 1 year, and higher daily doses of prednisone were independently associated with a 2- to 3-fold increased recurrence rate. This suggests that pericarditis recurrence reflects patient characteristics and treatment choices. The association between SLEDAI score and risk of recurrence had a lower RR when we removed pericarditis from the calculation of the SLEDAI score. We hypothesize that this may simply reflect a loss of statistical power. Overall, our findings suggest that specific subgroups of patients with pericarditis may benefit from close monitoring and early initiation of therapies proven to be effective for recurrent pericarditis, especially given that recurrence is more likely to occur within 1 year of the onset of pericarditis.^23^

Ryu et al^3^ demonstrated that 1 factor associated with acute pericarditis was African American race. In our study, we demonstrated an RR of 1.72 (95% CI, 0.99-2.97) for Black race and risk of recurrence, although this was not a statistically significant outcome. Kallas et al^24^ further validated the concerning finding that Black patients with SLE were more likely to experience worse clinical outcomes. Given that this observation was overall in line with our findings, further investigation will be needed to understand the root cause of the racial difference we highlighted.^25^

The medical management of pericarditis for patients with SLE differs from that for the general population. Although parenteral administration of glucocorticoids may be preferred to prolonged oral administration to mitigate the risk of complications (eg, avascular necrosis), oral glucocorticoids are often still considered for the treatment of SLE flares, including pericarditis.^26,27^ In contrast, colchicine and nonsteroidal anti-inflammatory drugs are preferred first-line agents for the treatment of acute pericarditis in the general population.^4,28,29^ In fact, the Investigation on Colchicine for Acute Pericarditis (ICAP) trial demonstrated that glucocorticoid use was an independent risk factor for recurrence.^29^ Furthermore, a meta-analysis of 7 studies that included 471 patients^30^ confirmed these findings; not only were steroids associated with increased risk of recurrent pericarditis, but low-dose steroids were also associated with lower odds of recurrence compared with high-dose steroids (odds ratio, 0.29; 95% CI, 0.13-0.66). In the Hopkins Lupus Cohort, we observed a dose-related association of oral prednisone treatment with risk of pericarditis recurrence similar to that in the general population, which was confirmed in multivariable analysis. This suggests that oral prednisone should be avoided whenever possible in patients with SLE and a history of pericarditis. Further studies will be needed to understand the role of colchicine and interleukin 1–blocking antibodies in treating recurrent pericarditis in patients with SLE.

### Limitations

The retrospective analysis of this cohort study led to several limitations. First, recurrent pericarditis was defined as that occurring at least 6 weeks from the last recorded episode of pericarditis, in accordance with the European Society of Cardiology.^20^ However, given the retrospective nature of this study, there is a chance that some episodes of incessant pericarditis may have been incorrectly defined as recurrent. Given that patients with persistent symptoms are typically reevaluated in short course in our clinical practice, the likelihood that incessant pericarditis was inappropriately defined is very low.

Second, SELENA-SLEDAI criteria were used to diagnose pericarditis. The SELENA-SLEDAI definition of pericarditis is less stringent than that used by cardiology associations, such as the European Society of Cardiology, as well as other forms of the SLEDAI criteria, including SLEDAI-2K.^20,26,31^ According to the SELENA-SLEDAI definition, only 1 of the following criterion must be present for diagnosis: pericardial pain, auscultation of pericardial rub, presence of pericardial effusion on imaging, or ECG confirmation.^17,20^ Within our study cohort, patient-reported pericardial chest pain was used most often to diagnose pericarditis. Importantly, we found that 21 patients who were further evaluated via specific testing, including transthoracic echocardiogram, computed tomography chest imaging, or ECG, demonstrated findings indicative of pericarditis. They thereby fulfilled both SLEDAI and European Society of Cardiology criteria for the diagnosis of pericarditis. This suggests that clinical criteria alone may be a useful tool for diagnosing pericarditis among patients with SLE.

## Conclusions

In this cohort study of patients with SLE and a history of pericarditis, we described the rate of recurrent pericarditis among patients with SLE and risk factors associated with recurrence. Our data suggest that despite the common practice to use prednisone to treat SLE flares, the use of oral corticosteroids should be avoided for patients with a recent history of pericarditis. Future studies within this unique population will be needed to determine the most effective treatment method for pericarditis, the most common cardiac complication of systemic lupus erythematosus.

## Notes

Supplement 1. **eTable 1.** Sensitivity analysis regarding rates of recurrent pericarditis and disease activity **eTable 2.** Factors associated with recurrence of pericarditis using SLEDAI score that excludes diagnosis of pericarditis **eFigure.** Study flowchart

Supplement 2.
Data Sharing Statement
