# Supplementary material for: Incidence and Factors Associated With Recurrent Pericarditis in Lupus
Source: JAMA Netw Open. 2025 Feb 25;8(2):e2461610. doi: 10.1001/jamanetworkopen.2024.61610 (PMC11862964; doi:10.1001/jamanetworkopen.2024.61610)
Supplement: Supplement 1. — eTable 1. Sensitivity analysis regarding rates of recurrent pericarditis and disease activity eTable 2. Factors associated with recurrence of pericarditis using SLEDAI score that excludes diagnosis of pericarditis eFigure. Study flowchart [file jamanetwopen-e2461610-s001.pdf]

# Supplemental Online Content

Kim YJ, Lovell J, Diab A, et al. Incidence and factors associated with recurrent pericarditis in lupus. *JAMA Netw Open*. 2025;8(2):e2461610. doi:10.1001/jamanetworkopen.2024.61610

**eTable 1.** Sensitivity analysis regarding rates of recurrent pericarditis and disease activity

**eTable 2.** Factors associated with recurrence of pericarditis using SLEDAI score that excludes diagnosis of pericarditis

**eFigure.** Study flowchart

This supplemental material has been provided by the authors to give readers additional information about their work.

© 2025 Kim YJ et al. *JAMA Network Open*.

**Supplemental Table 1. Sensitivity analysis regarding rates of recurrent pericarditis & disease activity**

| Exposure | Events, no. | Rate, per person-years | Rate ratio (95% CI) |
|---|---|---|---|
| Disease activity, SLEDAI[a] | | | |
| 0[b] | 61 | 0.038 | 1.0 (Ref) |
| 1-2 | 64 | 0.047 | 1.29 (0.88, 1.91) |
| **≥3** | **141** | **0.080** | **1.87 (1.33, 2.64)** |

[a] SLEDAI with the exclusion of pericarditis
[b] Reference rate for determination of rate ratio for the recurrence of pericarditis

**Supplemental Table 2. Predictors of recurrence of pericarditis using SLEDAI score that excludes the diagnosis of pericarditis**

| Predictor | Odds ratio. | 95% CI |
|---|---|---|
| Age, years | | |
| <40 | 1.0 | *Ref group* |
| 40-49 | 0.74 | 0.44, 1.26 |
| 50-59 | 0.38 | 0.19, 0.76 |
| **≥60** | **0.11** | **0.04, 0.30** |
| Time from initial episode, years | | |
| <1 | 1.0 | ***Ref group*** |
| 1-3 | 0.64 | 0.40, 1.03 |
| **3-10** | **0.31** | **0.19, 0.51** |
| **≥10** | **0.26** | **0.13, 0.53** |
| Prednisone dose, mg/day | | |
| 0 | 1.0 | *Ref group* |
| 1-9 | 1.17 | 0.70, 1.97 |
| 10-19 | 1.36 | 0.75, 2.45 |
| **≥20** | **2.13** | **1.25, 3.63** |
| SLEDAI score | | |
| <3 | 1.0 | *Ref group* |
| ≥3 | 1.24 | 0.96, 1.61 |
| **History of nephrotic syndrome** | **0.44** | **0.25, 0.77** |

© 2025 Kim YJ et al. *JAMA Network Open*.

**Supplemental Figure.** Consolidated Standards of Reporting Trials (CONSORT) diagram of patient selection criteria from the Hopkins Lupus Cohort.

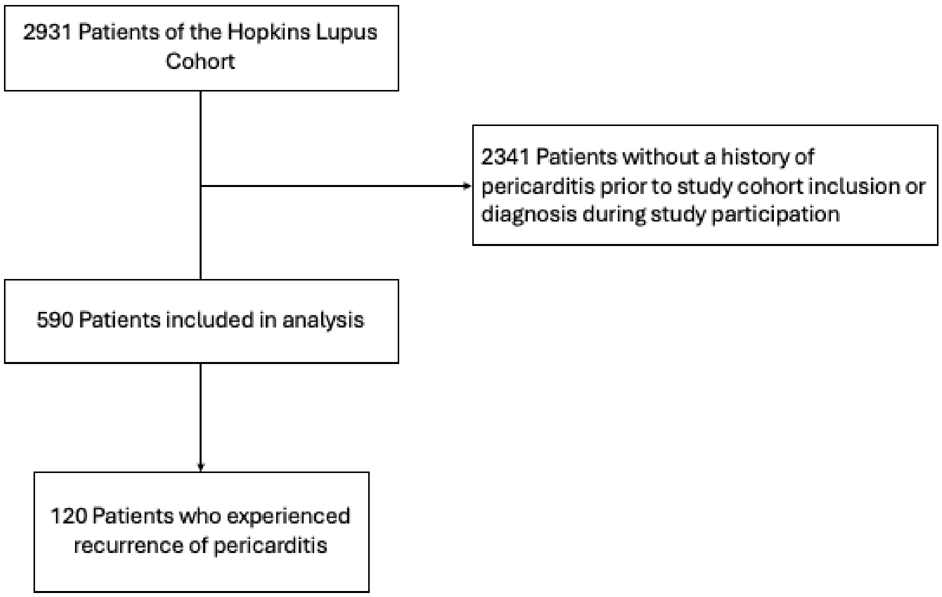


© 2025 Kim YJ et al. *JAMA Network Open*.
