# Supplementary material for: Incidence and Factors Associated With Recurrent Pericarditis in Lupus
Source: JAMA Netw Open. 2025 Feb 25;8(2):e2461610. doi: 10.1001/jamanetworkopen.2024.61610 (PMC11862964; doi:10.1001/jamanetworkopen.2024.61610)
Supplement: Supplement 2. — Data Sharing Statement [file jamanetwopen-e2461610-s002.pdf]

Data Sharing Statement

Kim. Incidence and Factors Associated With Recurrent Pericarditis in Lupus. *JAMA Netw Open*. Published online February 25, 2025. doi:10.1001/jamanetworkopen.2024.61610

Data
Data available: No
